# Malignant transformation of meningioma: Case report

**DOI:** 10.1097/MD.0000000000033409

**Published:** 2023-03-31

**Authors:** Xiaoqin Qu, Jingcheng Jiang, Han Wang, Chao Zhang, Qingshan Deng, Xiaoping Xu, Shijun Zhou, Yong Yi, Lihua Qiu

**Affiliations:** a Department of Radiology, The Affiliated Hospital of Southwest Medical University, Luzhou, China; b Department of Radiology, The Second People’s Hospital of Yibin, Clinical Research and Translational Center, Neuroimaging Big Data Research Center, The Second People’s Hospital of Yibin, Yibin, China; c Department of Neurosurgery, The Second People’s Hospital of Yibin, Yibin, China.

**Keywords:** anaplastic meningioma, case report, meningioma

## Abstract

Meningiomas are mostly benign brain tumors with minimal malignant cases. Anaplastic meningioma has malignant morphological characteristics and a World Health Organization grade of III.

**Patient concerns:**

The present study reports a case of an occipital meningioma in a patient who initially chose observation and follow-up after diagnosis. The patient ultimately underwent surgery due to the enlargement of the tumor and the development of visual field defects after a decade of imaging follow-up. The postoperative pathology slides indicated the presence of an anaplastic meningioma (World Health Organization-grade III).

**Diagnoses:**

The patient’s diagnosis was established through cranial magnetic resonance imaging, which revealed an irregular mixed mass in the right occipital region with isointense T1 and hypointense T2 signal, irregular lobulation, and a maximum diameter of approximately 5.4 cm. Heterogenous enhancement was observed in the contrast-enhanced scan.

**Interventions:**

The patient opted for surgical intervention to remove the tumor, and the pathology slides of the tumor sample confirmed the diagnosis of anaplastic meningioma. The patient also received radiotherapy (40Gy/15fr).

**Outcomes:**

No recurrence was observed during the 9-month follow-up.

**Lessons:**

This case highlights the potential for low-grade meningiomas to undergo malignant transformation, particularly in the presence of irregular lobulation, peritumoral brain edema, and heterogeneous enhancement on contrast-enhanced scans. Total excision (Simpson grade I) is the preferred treatment option, and long-term imaging follow-up is recommended.

## 1. Introduction

Meningiomas are a prevalent type of brain tumor, with the majority of cases being benign in nature.^[1]^ The present study reports a case of an occipital meningioma in a patient who initially elected for a strategy of observation and monitoring following the initial diagnosis. However, due to the enlargement of the tumor and the emergence of visual field defects over a period of 10 years of imaging follow-up, the patient ultimately underwent surgical intervention. The postoperative pathology analysis revealed the presence of anaplastic meningioma (World Health Organization [WHO]-grade III). To the best of our knowledge, this represents a case of anaplastic meningioma with the longest documented imaging follow-up interval since the initial diagnosis. While there is currently no consensus on the time frame for malignant transformation in low-grade meningiomas, it is acknowledged that there is a risk of malignant transformation.^[2]^ Therefore, it is recommended that patients receive intervention as soon as possible following the identification of a space-occupying lesion at the initial imaging diagnosis. Total excision is considered the preferred treatment option, and long-term imaging follow-up is required postoperatively.

## 2. Case presentation

**Figure 1.**
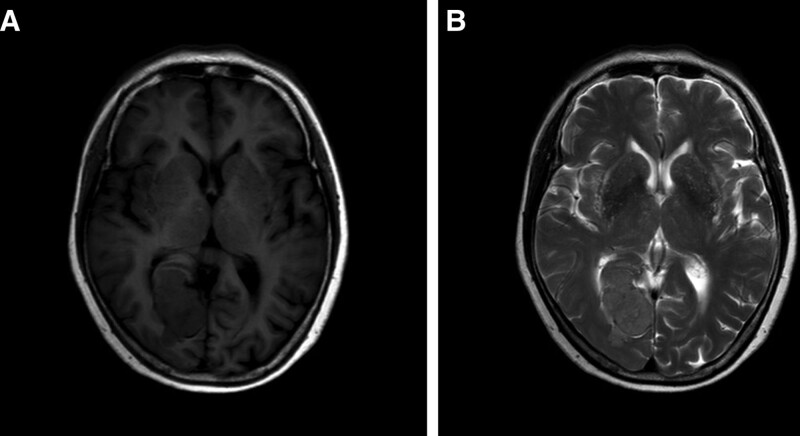
Cranial MRI in 2012: an oval like mass in the right occipital region with isointense on T1WI (A) and T2WI (B) without peritumoral edema, and maximum diameter about 3 cm. The posterior horn of the right ventricle is compressed. MRI = magnetic resonance imaging.

**Figure 2.**
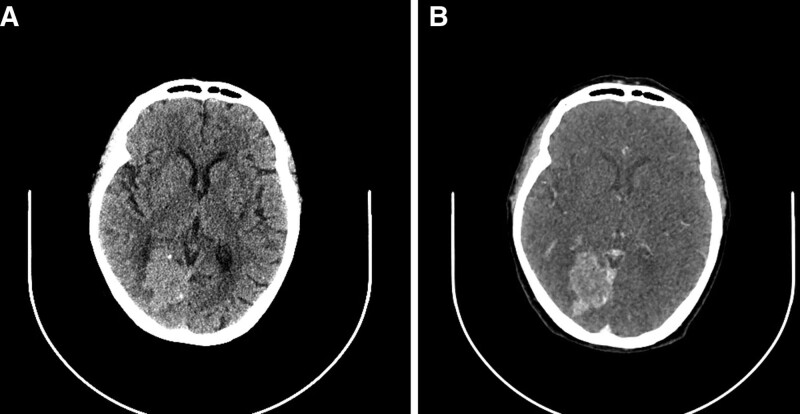
Cranial CT in 2017. (A) An irregular isodense mass in the right occipital region and dotted calcification were found at the edge of the mass. (B) Moderately homogeneous enhancement after contrast materials injected and maximum diameter of the mass is 4.5 cm. CT=computed tomography.

**Figure 3.**
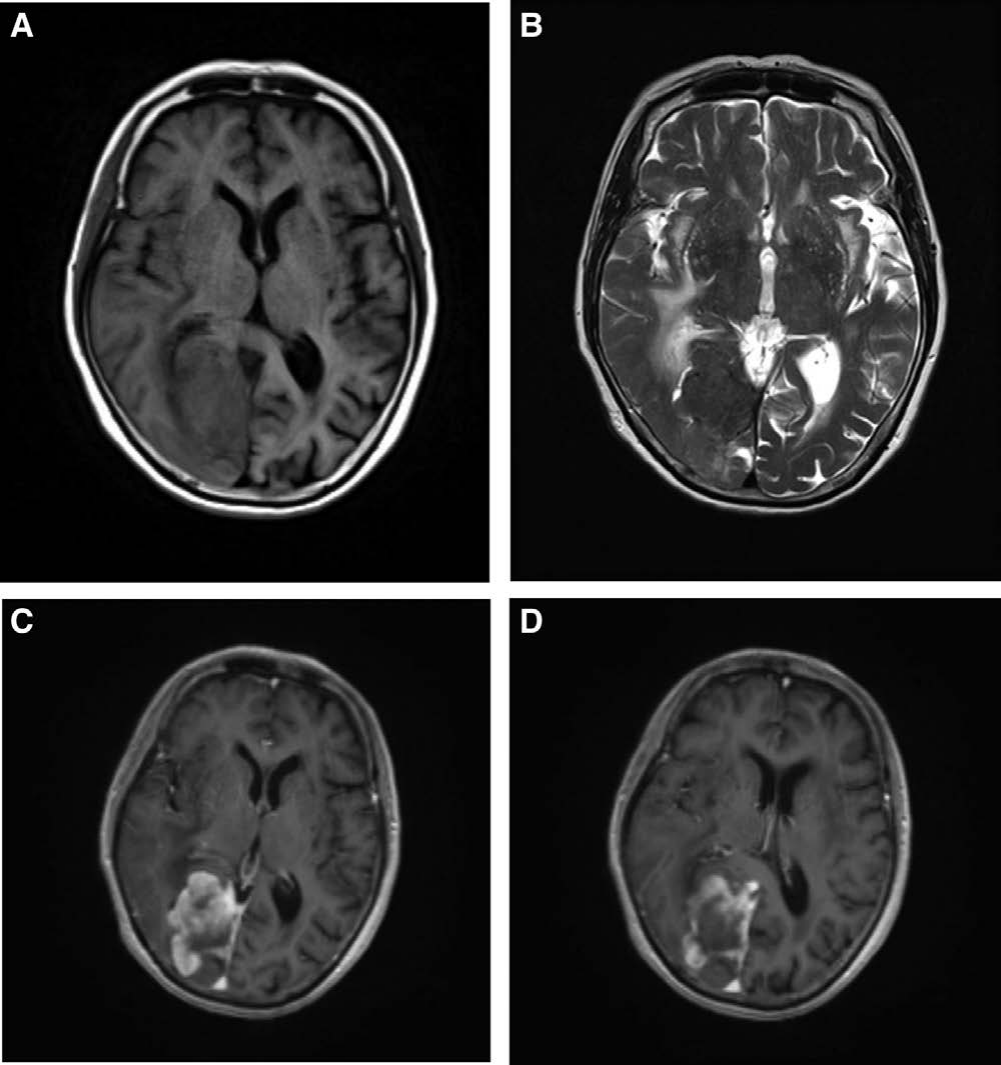
Cranial MRI in 2022: an irregular lobulated mass with moderate peritumoral edema in the right occipital region with a maximum diameter of 5.4 cm; heterogeneous enhancement after contrast-enhanced scan (C and D). MRI = magnetic resonance imaging.

The present study reports on a case of an occipital meningioma in an 80-year-old female patient. In 2012, a space-occupying lesion was identified in the right occipital region with an elliptical shape measuring approximately 3 cm in diameter. Based on imaging, the diagnosis of meningioma was considered (Fig. 1). The patient elected to not undergo surgical resection due to the absence of neurologic symptoms. No radiotherapy was administered, and the patient was monitored using imaging. In 2017, a cranial computed tomography scan revealed that the tumor had enlarged and exhibited an irregular shape with a maximum diameter of approximately 4.5 cm. The patient elected to continue observation using imaging (Fig. 2). In January 2022, the patient presented with visual field defects and sought medical attention. Neurological examination revealed left visual field defects in both eyes. Laboratory evaluations, including hematological indices, coagulation, renal function, hepatic function, and electrocardiogram were within normal ranges. Cranial magnetic resonance imaging revealed an irregular mixed mass in the right occipital region beside the cerebral falx with isointense T1 and hypointense T2 signal, irregular lobulation, and a maximum diameter of approximately 5.4 cm. Heterogenous enhancement was observed on the contrast-enhanced scan, and significant brain edema was present at the perimeter of the tumor (Fig. 3).

## 3. Treatment

After conducting a thorough consultation with the patient and her surrogates, the decision was made to proceed with surgical intervention to remove the tumor. The tumor, located in the right occipital region, was determined to have originated from the tentorium cerebelli and posterior falx. Upon incision of the dura, significant brain tissue edema was observed. The tumor presented as a solid, grayish-white, irregularly lobulated mass with a lack of abundant blood supply. The interface between the tumor and the normal brain tissue arachnoid mater was indistinct, and the tumor was identified as a meningioma through visual examination. The tumor was entirely excised, along with the tumor base at the tentorium cerebelli and cerebral falx, under microscope guidance (Simpson grade I).

**Figure 4.**
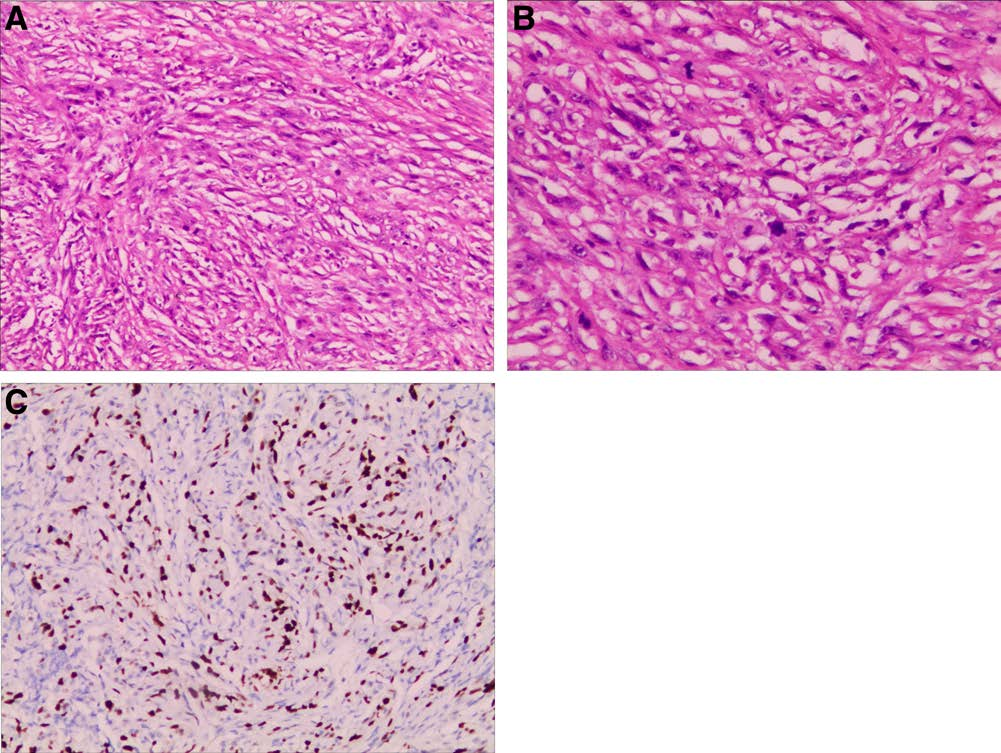
The histopathological section was consistent with anaplastic meningioma. Hematoxylin and eosin: (A) magnification 100×, (B) magnification 200×; Immunohistochemisty: (C) Ki-67 labeling index is 35%.

The pathology analysis of the tumor sample revealed a predominance of atypical spindle tumor cells with extensive multifocal necrosis and partial readily-visible nuclear division. Immunohistochemistry results were PR (partial +), EMA (focal +), S-100 (small focal +), CD34 (partial +), P53 (partial +), and Ki-67 (+, approximately 35% of hot spots). Collectively, this led to the diagnosis of anaplastic meningioma (WHO-grade III) (Fig. 4).

Following postoperative treatment, the patient’s visual field defects improved. The patient also received radiotherapy (40Gy/15fr) and there was no evidence of recurrence during the 9-month follow-up period.

## 4. Discussion

Meningiomas are tumors that arise from arachnoid cap cells and are among the most prevalent intracranial tumors, often observed in regions with abundant arachnoid granulations.^[1]^ According to the 2021 WHO classification system, central nervous system tumors are classified into 15 subtypes under grades I, II, and III.^[3]^ The majority of meningiomas are histologically benign and can be effectively treated through surgical resection. Atypical (WHO-grade II) and anaplastic meningiomas (WHO-grade III) have a tendency to recur.^[4]^ Anaplastic meningiomas (WHO-grade III) can be broadly categorized into 2 groups: cases diagnosed by pathology slides during the initial surgery, and cases that have transformed from low-grade meningiomas.^[2]^ The 5-year survival rate of anaplastic meningiomas (WHO-grade III) ranges from 8 to 61%.^[5]^ In the current case, the interval from initial imaging diagnosis to surgery was 10 years, indicating a slow progression. It is plausible to suspect that the anaplastic meningioma (WHO-grade III) in this case was transformed from a low-grade meningioma (WHO-grade I). Factors that may contribute to this transformation include radiotherapy, surgical stimulation, viral and chromosomal variations, patient age, and tumor location.^[6]^ Imaging findings, in this case, revealed an irregularly lobulated tumor with peritumoral brain edema, and heterogeneous enhancement on contrast-enhanced scans, which are typically indicative of malignancy.^[7]^ Total tumor resection (Simpson grade I) and adjuvant radiotherapy have been shown to be beneficial in extending survival.^[8]^

## 5. Conclusion

There is currently no consensus on the time frame for the malignant transformation of meningiomas. This particular case, with a 10-year follow-up period, represents one of the longest follow-up periods reported in the literature and serves as a valuable reference in the field of neuro-oncology. However, even after the malignant meningioma is removed at a Simpson grade I level, recurrence may still occur with adjuvant radiotherapy. Therefore, it is recommended that intervention be initiated as soon as possible upon the initial diagnosis of meningioma. Total excision (Simpson grade I) is the preferred course of treatment, and long-term imaging follow-up is recommended. For patients who opt for imaging follow-up after the initial diagnosis, a trend of tumor enlargement should prompt consideration of further intervention, particularly in cases that present with irregular lobulation, peritumoral brain edema, and heterogeneous enhancement on contrast-enhanced scans, as these may indicate malignant transformation.

## Author contributions

**Conceptualization:** Xiaoqin Qu, Jingcheng Jiang, Lihua Qiu, Yong Yi.

**Data curation:** Xiaoqin Qu, Jingcheng Jiang, Han Wang, Chao Zhang, Qingshan Deng, Xiaoping Xu, Shijun Zhou.

**Formal analysis:** Jingcheng Jiang.

**Investigation:** Jingcheng Jiang.

**Supervision:** Lihua Qiu, Yong Yi.

**Validation:** Yong Yi.

**Writing – original draft:** Xiaoqin Qu, Jingcheng Jiang, Han Wang, Chao Zhang, Qingshan Deng, Xiaoping Xu, Shijun Zhou.

**Writing – review & editing:** Lihua Qiu, Yong Yi.
